# Two‐Step Sequential Screening for Frailty in Patients With Chronic Liver Disease: A Triage Efficiency Study of Liver Frailty Index Components

**DOI:** 10.1002/nop2.70915

**Published:** 2026-10-19

**Authors:** Juan Wang, Shan Ouyang, Lihua Zheng, Shuxian Jiang, Na He, Lili Li

**Affiliations:** ^1^ Department of Infectious Diseases The Third Affiliated Hospital of Sun Yat‐Sen University Guangzhou Guangdong China

**Keywords:** chair stand test, frailty, hand strength, liver cirrhosis, liver diseases, liver frailty index, mass screening, nursing assessment

## Abstract

**Aim:**

To develop and validate a nurse‐administered two‐step sequential screening pathway based on single Liver Frailty Index components for identifying frailty in people with chronic liver disease.

**Design:**

A single‐centre cross‐sectional diagnostic accuracy study.

**Methods:**

Overall, 606 hospitalised patients with chronic liver disease were enrolled between August 2021 and August 2025. Timed chair‐stands and grip strength served as step‐one index tests against frailty defined by the Liver Frailty Index (score of 4.5 or higher); bedridden patients were counted as screen‐positive. Cutoffs were derived with the Youden index and stress‐tested with bootstrap resampling and split‐sample validation.

**Results:**

Frailty prevalence was 18.6%. The either‐positive rule (chair stands of 16.55 s or longer, or grip strength of 23.42 kg or lower) achieved 100% sensitivity and a negative predictive value of 1.000, missing zero frail patients, while sparing 62.3% of patients from the complete index assessment. Zero missed cases were reproduced in five split‐sample analyses.

**Conclusion:**

In this cohort, nearly two‐thirds of full assessments could have been avoided without missing LFI‐defined frailty cases. Prospective multicentre external validation of the frozen cutoffs is required before routine implementation.

**Implications for the Profession and/or Patient Care:**

The pathway may reduce the burden of complete frailty assessment in inpatient hepatology nursing practice, pending external validation.

**Impact:**

Universal frailty screening in chronic liver disease is hindered by the burden of completing the full index. In this cohort, the first step identified all LFI‐defined frailty cases while avoiding nearly two‐thirds of complete assessments. These findings matter to hepatology nurses and inpatient hepatology services seeking feasible, guideline‐concordant universal screening.

**Reporting Method:**

This study adhered to the Standards for Reporting of Diagnostic Accuracy Studies (STARD) 2015 guideline.

**No Patient or Public Contribution:**

Patients or the public were not involved in the design, conduct, reporting, or dissemination plans of this research.

## 1 Introduction

Frailty has emerged as a central determinant of outcomes in chronic liver disease (CLD). Once regarded primarily as a geriatric syndrome, it is now recognised as a prevalent and clinically consequential phenotype across the spectrum of cirrhosis severity, affecting a substantial proportion of patients well before they reach transplant evaluation (Xie et al. 2024). The evidence linking frailty to adverse outcomes is extensive and consistent: frailty independently predicts waitlist mortality in liver transplant candidates (Lai et al. 2014; Kardashian et al. 2021) remains associated with death after adjustment for ascites and hepatic encephalopathy in multicentre cohorts (Lai, Rahimi, et al. 2019) and is associated with accelerated cirrhosis disease progression (Wang et al. 2022). Importantly, frailty is not a fixed trait; longitudinal data indicate that changes in frailty over time track with subsequent mortality, implying a window for intervention if frailty is identified early (Lai et al. 2020). Reflecting this evidence base, the American Association for the Study of Liver Diseases, the American Society of Transplantation, and the European Association for the Study of the Liver all recommend routine frailty assessment as part of standard care for patients with cirrhosis (Lai, Tandon, et al. 2021; Lai, Sonnenday, et al. 2019; European Association for the Study of the Liver 2019). Guideline endorsement, however, has not translated into uniform bedside implementation.

The liver frailty index (LFI), a continuous composite of grip strength, repeated chair stands, and balance testing, is the most extensively validated instrument for quantifying frailty in this population and functions as the de facto reference standard (Lai et al. 2017). Yet completing all three performance‐based components imposes practical burdens: the full battery requires equipment, space, and several minutes per patient; critically ill, bedridden, or severely ascitic patients frequently cannot complete one or more components; and applying the full index to every patient in high‐volume wards and clinics is time‐intensive (Elsheikh et al. 2023). Questionnaire‐based alternatives are not a satisfactory substitute, as instruments such as SARC‐F have shown inadequate sensitivity in chronic liver disease populations (Hanai et al. 2021). These barriers are compounded in China, where LFI‐based data remain limited to a small number of single‐centre studies in outpatient and inpatient settings (Siyu et al. 2023) and no validated triage strategy exists to reconcile guideline‐mandated universal screening with real‐world clinical capacity.

Sequential, two‐step screening offers an established methodological solution to exactly this tension. In psychiatry, the ultra‐brief PHQ‐2 gates access to the full PHQ‐9, preserving sensitivity while reserving resource‐intensive assessment for screen‐positive patients (Kroenke et al. 2003); in geriatric medicine, simple performance measures have been systematically evaluated as first‐stage screens for frailty (Clegg et al. 2015) and a pragmatic two‐step algorithm for sarcopenia and frailty in community‐dwelling older adults was recently validated (Aliberti et al. 2026). Within hepatology, several adjacent efforts have sought to lighten the frailty‐assessment burden: a telemedicine‐adapted frailty instrument (TeLeFI) (Wang et al. 2023) a brief self‐reported PROMIS screen (Thuluvath et al. 2024) prognostic validation of a single chair‐stand component (Nguyen et al. 2023) and head‐to‐head comparison of five parallel screening tools against the LFI (Geladari et al. 2024). None of these studies, however, addressed the workflow question directly: each proposed an alternative instrument, a parallel comparison, or an isolated component‐outcome association, rather than testing whether the LFI's own rapid components can serve as a first‐stage gate—reserving the complete LFI, as the confirmatory reference standard, for screen‐positive patients—and quantifying the safety and efficiency of such a sequential pathway.

The present study was designed to fill this gap. In a cross‐sectional cohort of 606 hospitalised patients with CLD, we developed and internally validated a nurse‐administered, bedside two‐step pathway in which a timed chair stands or grip strength screen (with bedridden status counted as screen‐positive) triages patients to the complete LFI. Our specific contributions are fourfold. First, we provide a quantitative evaluation of an LFI‐component sequential screening pathway, showing that an ‘either‐positive’ rule achieved zero missed frailty cases (sensitivity 100%, 95% CI: 96.8%–100%) while restricting the full LFI to 37.7% of patients, thereby sparing 62.3% of assessments; because the index tests are components of the reference standard, these results are framed as pathway and triage efficiency rather than independent diagnostic accuracy. Second, we elucidate the complementary capture mechanism underlying this observed zero‐miss result and demonstrate the robustness of frozen cut‐offs across temporal and odd–even split validation subsets. Third, we examine pathway strata against gradients in activities of daily living, fall risk, nutritional risk, and psychological distress, and we state its boundary honestly: the pathway targets LFI‐defined frailty (LFI ≥ 4.5), not pre‐frailty. Fourth, by relying exclusively on tests executable by nursing staff at the bedside within minutes, the pathway offers a potential route to guideline‐concordant frailty screening in routine hepatology practice, pending external validation.

The remainder of this paper is organised as follows: Section 2 describes the study design and analyses, Section 3 reports the results, and Section 4 discusses implications, limitations and future directions.

## 2 Materials and Methods

### 2.1 Study Design and Participants

This was a single‐centre cross‐sectional diagnostic pathway efficiency study, conducted and reported in accordance with the Standards for Reporting of Diagnostic Accuracy Studies (STARD) 2015 statement and its explanation and elaboration document (Bossuyt et al. 2015; Cohen et al. 2016). We consecutively enrolled patients with chronic liver disease (CLD) who underwent clinical frailty assessment at a tertiary hospital in southern China between August 2021 and August 2025. All participants were hospitalised patients; no outpatients were included. No disease‐severity restriction was applied; the cohort therefore included a substantial proportion of patients with advanced disease.

Inclusion criteria were: (1) age 18 years or older; (2) a confirmed clinical diagnosis of CLD, including chronic hepatitis and cirrhosis at either the compensated or decompensated stage; (3) ability to cooperate with the physical performance assessments; and (4) provision of written informed consent. Exclusion criteria were: (1) inability to cooperate with the assessments, or critical illness that rendered physical performance testing unsafe; (2) overt upper‐limb dysfunction, neuromuscular disease, or any other condition compromising the accuracy of grip strength measurement; and (3) missing key outcome variables. Patients confined to bed were not excluded: those unable to complete the timed chair stands test because of bed rest were retained and automatically classified as step‐one positive under the pre‐specified safety rule described below. These criteria match those of the parent cohort from which this analysis was derived. The study protocol was approved by the Medical Ethics Committee of the Third Affiliated Hospital of Sun Yat‐sen University (approval No. [2021]02‐060‐01; approved 13 April 2021), and all participants provided written informed consent. The flow of participants through the study and the derivation of each analysis set are shown in Figure 1.

**FIGURE 1:**
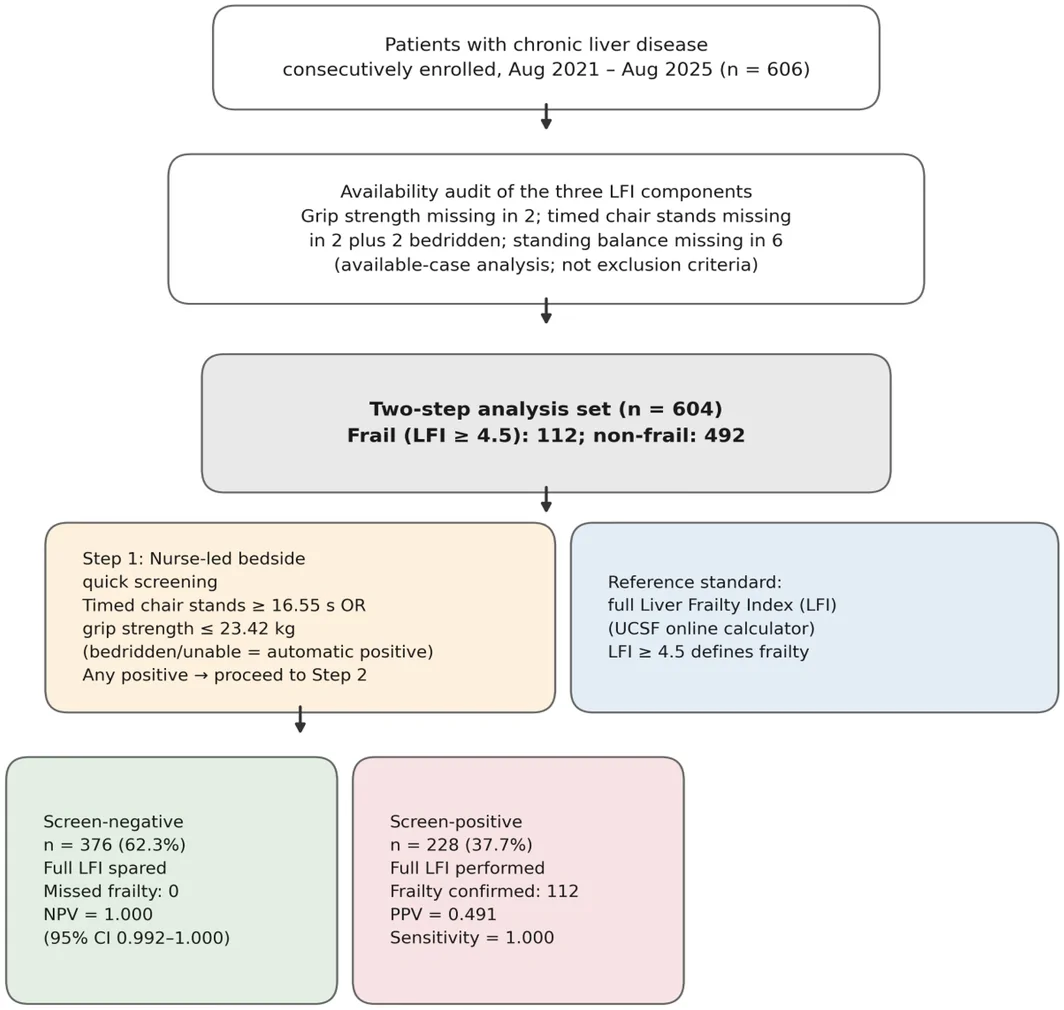
STARD study flow and the two‐step sequential screening pathway. LFI, liver frailty index; NPV, negative predictive value; PPV, positive predictive value.

#### 2.1.1 Component‐Level Missing Data

An item‐level audit of the 606 enrolled patients showed that grip strength was missing in 2 patients, timed chair stands (five‐repetition sit‐to‐stand) was missing in 2 patients and recorded as ‘bedridden’ in a further 2, and standing balance was missing in 6 patients. Liver frailty index (LFI) total scores and the binary frailty outcome were complete for all 606 patients. Because missingness was sparse and non‐systematic, we used available‐case analysis: the two‐step pathway analysis set comprised 604 patients (112 with LFI‐defined frailty and 492 without), and a complete‐component sensitivity analysis set comprised the 600 patients with all three LFI‐components measured. As a pre‐specified clinical safety rule, patients unable to attempt the timed chair stands test because of bed rest were automatically classified as step‐one positive, ensuring that inability to test triggered—rather than bypassed—further evaluation. No imputation was performed, because model‐based substitution of unmeasured physical performance values would introduce unverifiable assumptions.

### 2.2 Index Tests (Candidate Step‐One Tests)

All assessments were performed during a single encounter by nurses who had received standardised training.

Grip strength (kg). Grip strength was measured with an electronic dynamometer following the standardised approach recommended by (Roberts et al. 2011): the patient was seated whenever clinically feasible (a safe bedside position was permitted otherwise), with the shoulder adducted, the elbow flexed to approximately 90 degrees, and the forearm in a neutral position. Three trials were performed with the dominant hand at intervals of approximately 30–60 s, and the average value was recorded; lower values indicate poorer muscle function. In the interest of transparent reporting, we note that the analysis database and archived study records did not retain the brand, model, nominal resolution, or serial calibration records of the dynamometer; it can only be confirmed that routine functional checks and periodic maintenance were performed in accordance with the department's medical‐device management policy. Device metrological information is therefore not fully traceable and is reported as a reproducibility limitation.

#### 2.2.1 Timed Chair Stands (Seconds)

The timed chair stands test recorded the time required to rise five times from an armless chair with the arms crossed over the chest, consistent with the measurement framework of the revised European consensus on sarcopenia and with current sit‐to‐stand measurement guidance (Cruz‐Jentoft et al. 2019; Park and Shin 2024); longer times indicate poorer lower‐limb function.

Standing balance (seconds). Standing balance was quantified as the sum of the durations for which three positions (feet side‐by‐side, semi‐tandem and tandem) could be maintained, each up to a maximum of 10 s (maximum total 30 s), as specified in the original LFI protocol (Lai et al. 2017); shorter total durations indicate poorer balance.

### 2.3 Reference Standard

The reference standard was LFI‐defined frailty. The three component measurements were entered into the online University of California, San Francisco (UCSF) LFI calculator to obtain a continuous LFI score (Lai et al. 2017; Wang et al. 2019). Following established practice, frailty was defined as LFI of 4.5 or higher, pre‐frailty as 3.2 ≤ LFI < 4.5 and robustness as LFI below 3.2 (Lai et al. 2017; Kardashian et al. 2021); accordingly, patients with LFI values between 4.4 and 4.5 were classified as pre‐frail under this study's definition. The primary reference standard was LFI of 4.5 or higher; LFI of 3.2 or higher (frailty plus pre‐frailty) was examined as a secondary reference standard in a sensitivity analysis.

Incorporation bias statement. The candidate step‐one tests are themselves constituent components of the reference standard. As Ransohoff and Feinstein demonstrated in their analysis of spectrum and incorporation bias, evaluating a test against a reference standard that contains the test mechanically inflates apparent agreement and precludes interpretation of the resulting area under the receiver operating characteristic curve (AUC) as independent diagnostic accuracy (Ransohoff and Feinstein 1978). Accordingly, this study is explicitly framed as an evaluation of the efficiency and observed missed‐case performance of a sequential triage pathway, not as validation of an independent diagnostic test. The primary outcomes are therefore the number of frail patients missed by step one, the negative predictive value (NPV) of a step‐one‐negative result, and the proportion of patients requiring the full LFI assessment. This framing follows the methodology established for sequential two‐stage screening with the two‐item and nine‐item Patient Health Questionnaire, in which a brief first‐stage screen embedded in the full instrument is used to rule out disease and to restrict full assessment to screen‐positive patients (Kroenke et al. 2003; Levis et al. 2020). This overlap may partly explain the observed sensitivity and NPV of 1.000 and the absence of missed LFI‐defined frailty cases; these are within‐cohort pathway estimates, not independent diagnostic accuracy or evidence of clinical safety.

#### 2.3.1 Blinding

No dedicated blinding procedure was implemented, and the source records did not document the order of the index‐test and reference‐standard assessments or the identity of the assessors; expectation bias arising from the same nurse having access to all of a patient's functional information therefore cannot be excluded. The pathway‐evaluation framework does not eliminate this potential bias.

### 2.4 Two‐Step Pathway Construction

Single‐item thresholds for step one were derived by maximising the Youden index against the primary reference standard (Youden 1950; Fluss et al. 2005) yielding data‐derived, Youden‐optimal candidate cutoffs of 23.42 kg or less for grip strength and 16.55 s or longer for timed chair stands. The primary cutoffs were derived from the full‐sample, and the split‐sample analyses described below served as stability validation of these data‐derived thresholds rather than as independent derivation. The standing‐balance item was evaluated separately as an additional candidate step‐one test. The two single items were then combined under an ‘either‐positive’ (OR) rule: patients positive on either item at step one proceeded to step two (full LFI assessment), whereas patients negative on both were classified as screen‐negative and exempted from further testing. The OR rule was chosen a priori to prioritise sensitivity in a triage context and because the two items capture complementary physiological domains (upper‐limb strength vs. lower‐limb function) with non‐overlapping failure modes. As rule‐out‐oriented comparators, we additionally evaluated high‐sensitivity thresholds chosen to meet pre‐specified sensitivity targets (≥ 0.95 and ≥ 0.98) for each single item, consistent with the rule‐out rationale for frailty screening instruments (Clegg et al. 2015).

### 2.5 External Anchor States

To examine the external clinical validity of the pathway strata, we assessed four contemporaneous clinical states that are not components of the LFI: (1) activities of daily living (ADL) limitation, defined as a Barthel Index below 100—a deliberately inclusive threshold, because in an admission triage context, any degree of dependence may trigger nursing evaluation; (2) high fall risk, defined as a Morse Fall Scale score of 45 or higher; (3) nutritional risk, defined as a Nutritional Risk Screening 2002 (NRS 2002) score of 3 or higher; and (4) depressive and anxiety symptoms, assessed contemporaneously as part of routine nursing evaluation. For these psychological anchors, the assessment instrument names and positive cutoffs were not standardised by name in the source records; we report this transparently. Because these assessments were performed as part of routine nursing care rather than as mandated study procedures, they were available for a subset of 455 patients. These anchors were used to test whether the three pathway strata (screen‐negative, screen‐positive but LFI‐negative, and confirmed frail) form a gradient of concurrent clinical burden.

### 2.6 Statistical Analysis

Continuous variables are summarised as medians with interquartile ranges and categorical variables as counts with percentages. Normality of continuous variables was assessed with the Shapiro–Wilk test; because the major continuous variables departed from normality, between‐group comparisons of baseline characteristics used the Mann–Whitney *U* test for continuous variables and the chi‐square test for categorical variables, with Fisher's exact test applied when expected cell counts were below 5. Discrimination of each index test against the reference standard was quantified using receiver operating characteristic (ROC) analysis, reporting the AUC with 95% confidence intervals (CIs) obtained from 2000 bootstrap replicates; sensitivity, specificity, positive predictive value (PPV), and NPV were calculated at the Youden‐derived cutoffs. AUCs of the timed chair stands and grip strength were compared as paired, correlated curves using the DeLong test on the 602 patients in whom both tests were available (DeLong et al. 1988) with the 95% CI for the AUC difference estimated from 2000 paired bootstrap replicates. Pathway efficiency outcomes were the number of frail patients missed, sensitivity and NPV with Clopper–Pearson exact 95% CIs, the proportion of patients requiring full LFI assessment, and the corresponding proportion of assessments saved.

The validation strategy comprised four elements: (1) a temporal split, in which cutoffs were derived in the first 400 enrolled patients, frozen, and then applied unchanged to the subsequent 204 patients; (2) an odd–even cross‐split, in which the cohort was halved by enrollment‐order parity and cutoffs were exchanged across halves; (3) bootstrap stability assessment (2000 replicates) of the zero‐missed‐case property of the OR rule; and (4) an exploratory grid search over joint cutoffs under a sensitivity constraint of 1.00, to determine the maximum specificity attainable and thereby assess whether the primary pathway approached overfitting. Pre‐planned supplementary analyses included sex‐stratified ROC analyses, re‐analysis against the secondary reference standard of LFI of 3.2 or higher, and repetition of the primary pathway analysis in the 600‐patient complete‐component set. Post hoc exploratory analyses applied the pooled OR‐rule cutoffs unchanged across age strata and available clinical severity proxies (Child–Pugh grade, ascites status and recorded decompensation); no subgroup‐specific cutoffs were re‐estimated. No a priori sample size calculation was performed; the sample comprised all consecutive eligible patients assessed during the study period, and inference emphasises effect estimates with 95% CIs. All analyses were performed in R version 4.5.3 (R Foundation for Statistical Computing, Vienna, Austria) using the pROC package version 1.19.0.1; two‐sided *p* values below 0.05 were considered statistically significant. Kimi (Moonshot AI; accessed during June–August 2026) was used to support statistical code drafting, English‐language editing, and manuscript formatting during the preparation of this work; AI‐generated code was reviewed by the authors and executed locally in R, and all resulting statistical outputs were checked against the source data. All AI‐assisted content was reviewed and edited by the authors, who take full responsibility for the integrity and accuracy of the final manuscript.

## 3 Results

### 3.1 Participant Flow and Baseline Characteristics

The participant flow is summarised in Figure 1. Between August 2021 and August 2025, 606 patients with chronic liver disease were enrolled at a tertiary hospital in southern China (inpatient setting). According to the liver frailty index (LFI), calculated with the UCSF online calculator (Lai et al. 2017), 100 patients (16.5%) were classified as robust (LFI < 3.2), 393 (64.9%) as pre‐frail (3.2 ≤ LFI < 4.5) and 113 (18.6%) as frail (LFI ≥ 4.5) (Kardashian et al. 2021). Audit of component completeness identified missing grip strength in 2 patients, missing timed chair stands in 2 patients (plus 2 bedridden patients unable to perform the test) and missing standing balance in 6 patients. The two‐step analytic sample therefore comprised 604 patients (112 frail, 492 non‐frail); the sensitivity analysis set with complete data on all three components comprised 600 patients. No adverse events occurred during the nurse‐administered assessments.

Baseline characteristics of the full cohort, stratified by frailty status, are presented in Table 1. Compared with non‐frail patients, frail patients were older (median 59.00 vs. 48.00 years, *p* < 0.001) and less frequently male (67.3% vs. 83.6%, *p* < 0.001). Frail patients had lower body mass index (BMI; 21.77 vs. 22.77 kg/m^2^, *p* = 0.005), dry BMI (20.55 vs. 21.70 kg/m^2^, *p* = 0.001), serum albumin (32.90 vs. 34.50 g/L, *p* = 0.012), and prealbumin (64.00 vs. 75.00 mg/L, *p* = 0.008), and higher Model for End‐stage Liver Disease (MELD; 18.77 vs. 16.35, *p* = 0.030) and MELD‐Na scores (20.69 vs. 17.18, *p* = 0.006). Child‐Pugh scores also differed significantly between groups (*p* = 0.005), although the median was 9.00 in both groups. Ascites was more prevalent among frail patients (64.6% vs. 51.3%, *p* = 0.014). Geriatric syndrome burdens were markedly higher in the frail group: activities of daily living (ADL) limitation (75.2% vs. 27.2%), high fall risk (70.8% vs. 30.8%) and nutritional risk (43.4% vs. 19.1%) (all *p* < 0.001). Total bilirubin, creatinine, international normalised ratio, serum sodium, hepatic encephalopathy, and gastrointestinal bleeding did not differ significantly between groups.

**TABLE 1:** Baseline characteristics of the full cohort (*n* = 606), stratified by frailty status (LFI ≥ 4.5).

Characteristic	Overall (*n* = 606)	Non‐frail (*n* = 493)	Frail (*n* = 113)	*p*
Age, years	50.00 (40.00–59.00)	48.00 (39.00–57.00)	59.00 (46.00–69.00)	< 0.001
Male sex, *n* (%)	488 (80.5)	412 (83.6)	76 (67.3)	< 0.001
BMI, kg/m^2^	22.62 (20.32–25.35)	22.77	21.77	0.005
Dry BMI, kg/m^2^	21.37 (19.12–24.17)	21.70	20.55	0.001
Albumin, g/L	34.30 (30.70–37.70)	34.50	32.90	0.012
Prealbumin, mg/L	70.00 (47.00–111.75)	75.00	64.00	0.008
Total bilirubin, μmol/L	78.75	74.10	88.30	0.417
Creatinine, μmol/L	62.55	62.00	67.00	0.292
INR	1.43	1.42	1.51	0.054
Serum sodium, mmol/L	139.00	139.00	139.00	0.563
MELD score	17.07 (11.18–22.86)	16.35	18.77	0.030
MELD‐Na score	17.58 (11.71–23.92)	17.18	20.69	0.006
Child‐Pugh score	9.00 (7.00–11.00)	9.00	9.00	0.005
Child‐Pugh class C, *n* (%)	240 (39.6)	186 (37.7)	54 (47.8)	0.062
Ascites, *n* (%)	326 (53.8)	253 (51.3)	73 (64.6)	0.014
Hepatic encephalopathy, *n* (%)	58 (9.6)	44 (8.9)	14 (12.4)	0.341
Gastrointestinal bleeding, *n* (%)	41 (6.8)	29 (5.9)	12 (10.6)	0.109
ADL limitation (Barthel < 100), *n* (%)	219 (36.1)	134 (27.2)	85 (75.2)	< 0.001
High fall risk (Morse ≥ 45), *n* (%)	232 (38.3)	152 (30.8)	80 (70.8)	< 0.001
Nutritional risk (NRS2002 ≥ 3), *n* (%)	143 (23.6)	94 (19.1)	49 (43.4)	< 0.001

*Note:* Data are median (interquartile range) or *n* (%). This table presents the full cohort of 606 patients; the two‐step pathway analysis set comprised 604 patients (492 non‐frail, 112 frail) after exclusion of 2 patients with missing grip strength. Table 5 uses this 604‐patient analysis set, so the frail‐group count for ADL limitation there is 84 (75.0%) versus 85 (75.2%) in this table, a difference attributable solely to the denominator.

Abbreviations: ADL, activities of daily living; BMI, body mass index; INR, international normalised ratio; LFI, liver frailty index; MELD, model for end‐stage liver disease; NRS2002, nutritional risk screening 2002.

### 3.2 Single‐Component Screening Performance

Table 2 summarises the performance of each LFI component used alone as an index test against the reference standard of LFI‐defined frailty (LFI ≥ 4.5); as each component is part of the reference standard, the area under the receiver operating characteristic curve (AUC) estimates should be read as within‐pathway concordance rather than independent diagnostic accuracy (see the footnote to Table 2). Timed chair stands (*n* = 602) yielded an AUC of 0.982 (95% CI: 0.970–0.991); at the Youden‐index‐derived cutoff (Youden 1950) of ≥ 16.55 s, sensitivity was 0.918 and specificity 0.955, with 9 frail patients missed. Grip strength (*n* = 604) yielded an AUC of 0.920 (95% CI: 0.895–0.943); at a cutoff of ≤ 23.42 kg, sensitivity was 0.955 and specificity 0.791, with 5 missed. Standing balance performed poorly (*n* = 600; AUC 0.627, 95% CI: 0.583–0.674; sensitivity 0.336 at a cutoff of ≤ 9.00 s, missing 73 of 110 frail patients) and was judged unsuitable as a stand‐alone initial screen. The receiver operating characteristic curves are displayed in Figure 2. In a DeLong comparison on the 602 patients with both tests (DeLong et al. 1988) timed chair stands significantly outperformed grip strength (*Z* = 4.4365, *p* = 9.14 × 10^−6^; AUC difference 0.062, 95% CI: 0.035–0.089).

**TABLE 2:** Performance of individual LFI components as single‐step screens against LFI ≥ 4.5.

Component	*n*	AUC (95% CI)	Cutoff	Sensitivity	Specificity	PPV	NPV	Positive, %	Missed frail, *n*
Grip strength, kg	604	0.920 (0.895–0.943)	≤ 23.42	0.955	0.791	0.510	0.987	34.8	5
Timed chair stands, s	602	0.982 (0.970–0.991)	≥ 16.55	0.918	0.955	0.821	0.981	20.4	9
Standing balance, s	600	0.627 (0.583–0.674)	≤ 9.00	0.336	0.912	0.462	0.860	13.3	73

*Note:* Components are constituents of the reference standard; estimates reflect pathway concordance, not independent diagnostic accuracy.

Abbreviations: AUC, area under the receiver operating characteristic curve; CI, confidence interval; NPV, negative predictive value; PPV, positive predictive value.

**FIGURE 2:**
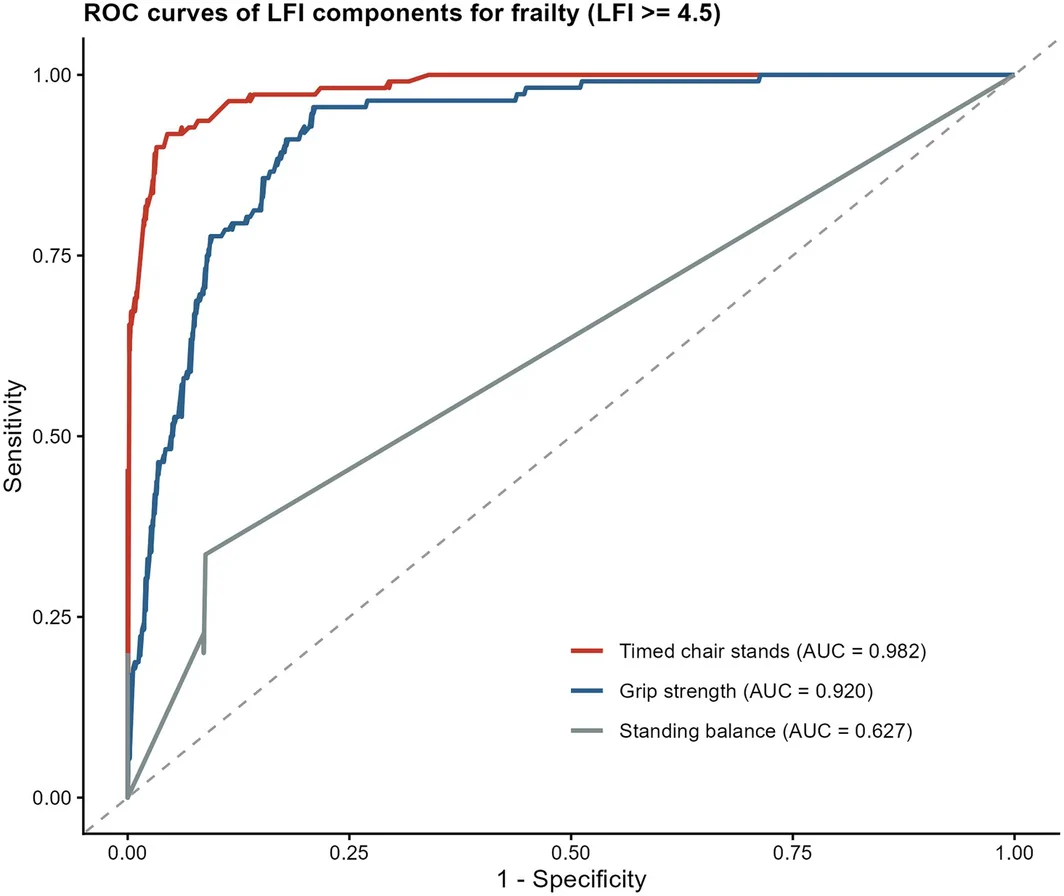
Receiver operating characteristic curves of the three liver frailty index components for identifying frailty (LFI ≥ 4.5). Estimates represent within‐pathway component–composite concordance, not independent diagnostic accuracy. AUC, area under the curve.

### 3.3 Two‐Step Pathway Simulation

Three triage strategies were simulated in the full analytic sample (*n* = 604), and the results are shown in Table 3. Timed chair stands alone (≥ 16.55 s) achieved sensitivity 0.920 and specificity 0.955, referring 125 patients (20.7%) to full LFI assessment and missing 9 frail patients. Grip strength alone (≤ 23.42 kg) achieved sensitivity 0.955 and specificity 0.791, referring 210 patients (34.8%) and missing 5. The OR‐rule combination (positive on either component) achieved sensitivity 1.000 (Clopper–Pearson 95% CI: 0.968–1.000), specificity 0.764, positive predictive value 0.491 and negative predictive value 1.000 (Clopper–Pearson 95% CI: 0.990–1.000; 376/376); 228 patients (37.7%) would proceed to full LFI assessment, sparing 62.3% of the cohort, with zero frail patients missed. The per‐100‐patient pathway is illustrated in Figure 3.

**TABLE 3:** Simulated two‐step triage strategies against LFI ≥ 4.5 (*n* = 604).

Strategy	Sensitivity	Specificity	PPV	NPV	Full LFI needed, *n*	Positive, %	Missed, *n*	Workload spared, %
Chair stands ≥ 16.55 s	0.920	0.955	0.824	0.981	125	20.7	9	79.3
Grip strength ≤ 23.42 kg	0.955	0.791	0.510	0.987	210	34.8	5	65.2
OR rule (either‐positive)	1.000	0.764	0.491	1.000	228	37.7	0	62.3

*Note:* Abbreviations as in Table 2. The analysis set (*n* = 604) includes 2 bedridden patients auto‐classified as step‐one positive under the pre‐specified safety rule; patients with unavailable component values were analysed as screen‐negative in the single‐item simulations, which accounts for the differing denominators between Table 2 (*n* = 602 for timed chair stands) and this table (*n* = 604).

**FIGURE 3:**
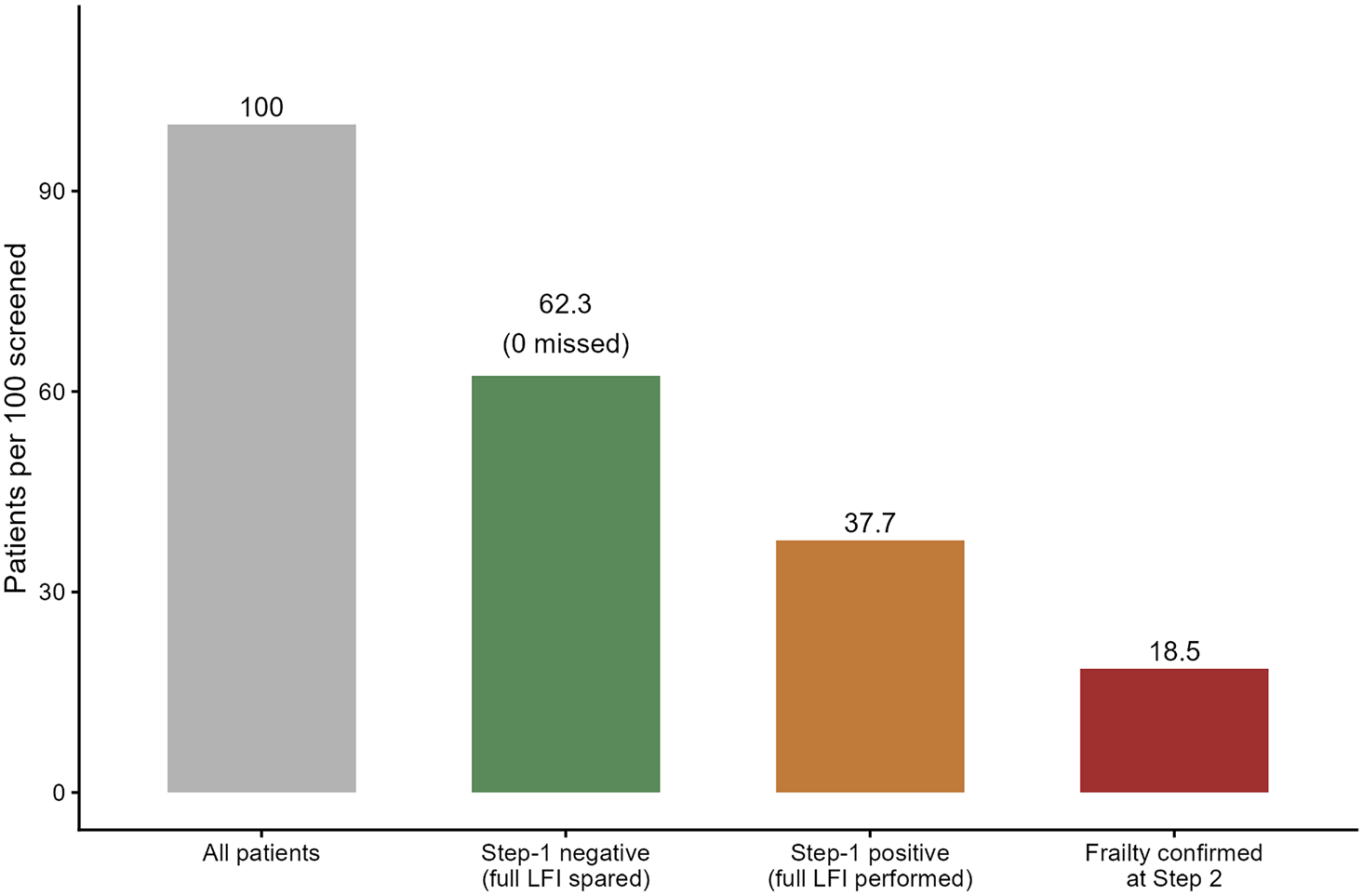
Population flow per 100 patients through the two‐step sequential screening pathway. In this cohort, 62.3% screened negative, with no missed LFI‐defined frailty cases.

Complementarity analysis of the 112 frail patients showed that 98 were positive on both components, 5 were positive only on timed chair stands, 9 were positive only on grip strength, and none was negative on both; the sets of patients missed by each single component were entirely non‐overlapping (the 5 frail patients missed by grip strength were exactly those positive only on timed chair stands and vice versa). In 2000 bootstrap resamples, the median sensitivity of the OR rule was 1.000 (both the 2.5th and 97.5th percentiles were 1.000), and zero missed cases occurred in 100% of resamples. Alternative high‐sensitivity rule‐out thresholds (e.g., chair stands ≥ 14.98 s: sensitivity 0.964, 4 missed) are reported in Table S1. An exploratory grid search under a sensitivity = 1.0 constraint identified a maximal achievable specificity of 0.770, close to the main‐strategy value of 0.764 (Figure 4).

**FIGURE 4:**
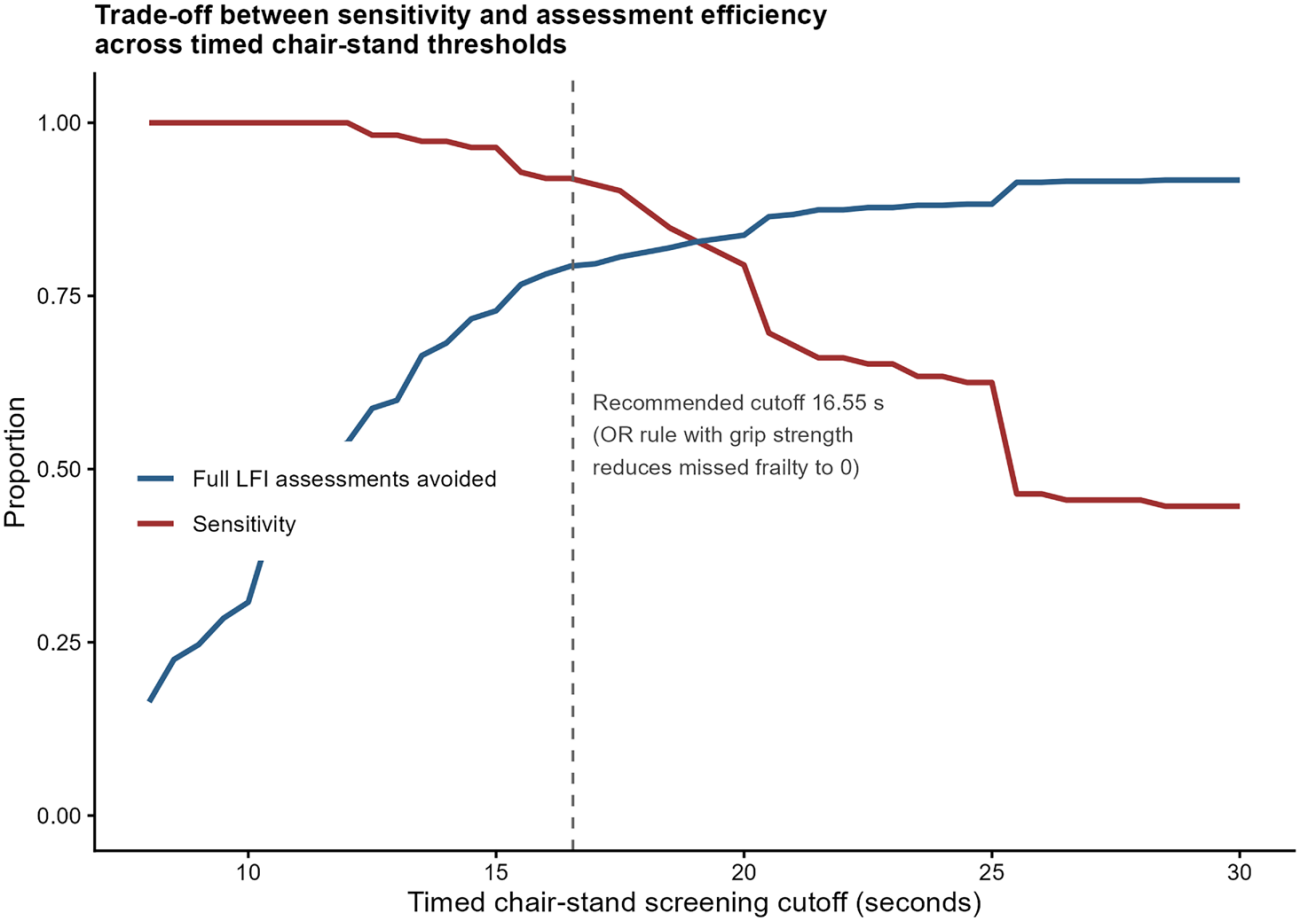
Trade‐off between sensitivity and the proportion of full LFI assessments avoided across timed chair‐stand thresholds. Dashed line indicates the recommended 16.55 s cutoff; adding the grip‐strength arm (OR rule) reduced missed frailty to zero.

### 3.4 Split‐Sample Validation

Split‐sample validation results are presented in Table 4. In the temporal split, the derivation set (first 400 enrolled patients; 86 frail) yielded cutoffs of ≥ 17.70 s for chair stands and ≤ 23.42 kg for grip strength, with in‐set sensitivity 1.000 and zero missed cases. After freezing these cutoffs, the independent temporal validation set (*n* = 204; 26 frail) showed sensitivity 1.000, specificity 0.803, negative predictive value 1.000, a positivity rate of 29.9%, and zero missed cases; applying the full‐sample cutoffs (16.55 s/23.42 kg) to the same validation set likewise yielded sensitivity 1.000 with zero missed cases (positivity 30.9%). Both directions of the odd–even cross‐split (*n* = 302 each) also achieved sensitivity 1.000 with zero missed cases. Cutoffs derived independently within each subset were highly consistent (chair stands 16.9–17.7 s; grip strength 23.2–23.4 kg).

**TABLE 4:** Split‐sample validation of the OR‐rule two‐step pathway.

Subset	*n*	Frail, *n*	Cutoffs (s/kg)	Sensitivity	Specificity	NPV	Positive, %	Missed, *n*
Temporal derivation	400	86	≥ 17.70/≤ 23.42	1.000	0.755	—	40.8	0
Temporal validation (frozen cutoffs)	204	26	≥ 17.70/≤ 23.42	1.000	0.803	1.000	29.9	0
Temporal validation (full‐sample cutoffs)	204	26	≥ 16.55/≤ 23.42	1.000	0.792	—	30.9	0
Odd–even split 1	302	64	≥ 16.9/≤ 23.4	1.000	0.773	—	—	0
Odd–even split 2	302	48	≥ 17.5/≤ 23.2	1.000	0.760	—	—	0

*Note:* Dashes indicate quantities not derived for that subset.

Abbreviation: NPV, negative predictive value.

### 3.5 External Anchor and Boundary Analyses

Table 5 displays the three‐stratum gradient of external geriatric and disease‐severity anchors across pathway‐defined groups: screen‐negative patients (*n* = 376, 62.3%), screen‐positive but LFI‐negative patients (*n* = 116, 19.2%), and confirmed frail patients (*n* = 112, 18.5%). The prevalence of ADL limitation rose stepwise from 25.0% to 34.5% to 75.0%; high fall risk from 26.9% to 43.1% to 70.5%; and nutritional risk from 15.4% to 30.2% to 42.9%. In the subset with available psychological assessments (*n* = 455; stratum denominators 279/84/92), depression increased from 21.9% to 22.6% to 33.7% and anxiety from 13.3% to 11.9% to 25.0%. Median LFI values were 3.57, 4.13, and 4.93 across the three strata. This gradient is visualised in Figure 5. Boundary analysis showed that 74.7% of screen‐negative patients (281/376) were pre‐frail by LFI, and the highest LFI among screen‐negative patients was 4.45.

**TABLE 5:** External anchor measures across pathway‐defined strata (*n* = 604).

Anchor	Screen‐negative (*n* = 376)	Screen‐positive, LFI‐negative (*n* = 116)	Confirmed frail (*n* = 112)
LFI, median	3.57	4.13	4.93
LFI, maximum	4.45	4.48	6.85
ADL limitation, *n* (%)	94 (25.0)	40 (34.5)	84 (75.0)
High fall risk, *n* (%)	101 (26.9)	50 (43.1)	79 (70.5)
Nutritional risk, *n* (%)	58 (15.4)	35 (30.2)	48 (42.9)
Depression, *n* (%)^a^	61/279 (21.9)	19/84 (22.6)	31/92 (33.7)
Anxiety, *n* (%)^a^	37/279 (13.3)	10/84 (11.9)	23/92 (25.0)
Child‐Pugh class C, *n* (%)	134 (35.6)	52 (44.8)	53 (47.3)

Abbreviations: ADL, activities of daily living; LFI, liver frailty index.

^a^ Depression and anxiety were assessed in 455 patients (stratum denominators 279, 84 and 92).

**FIGURE 5:**
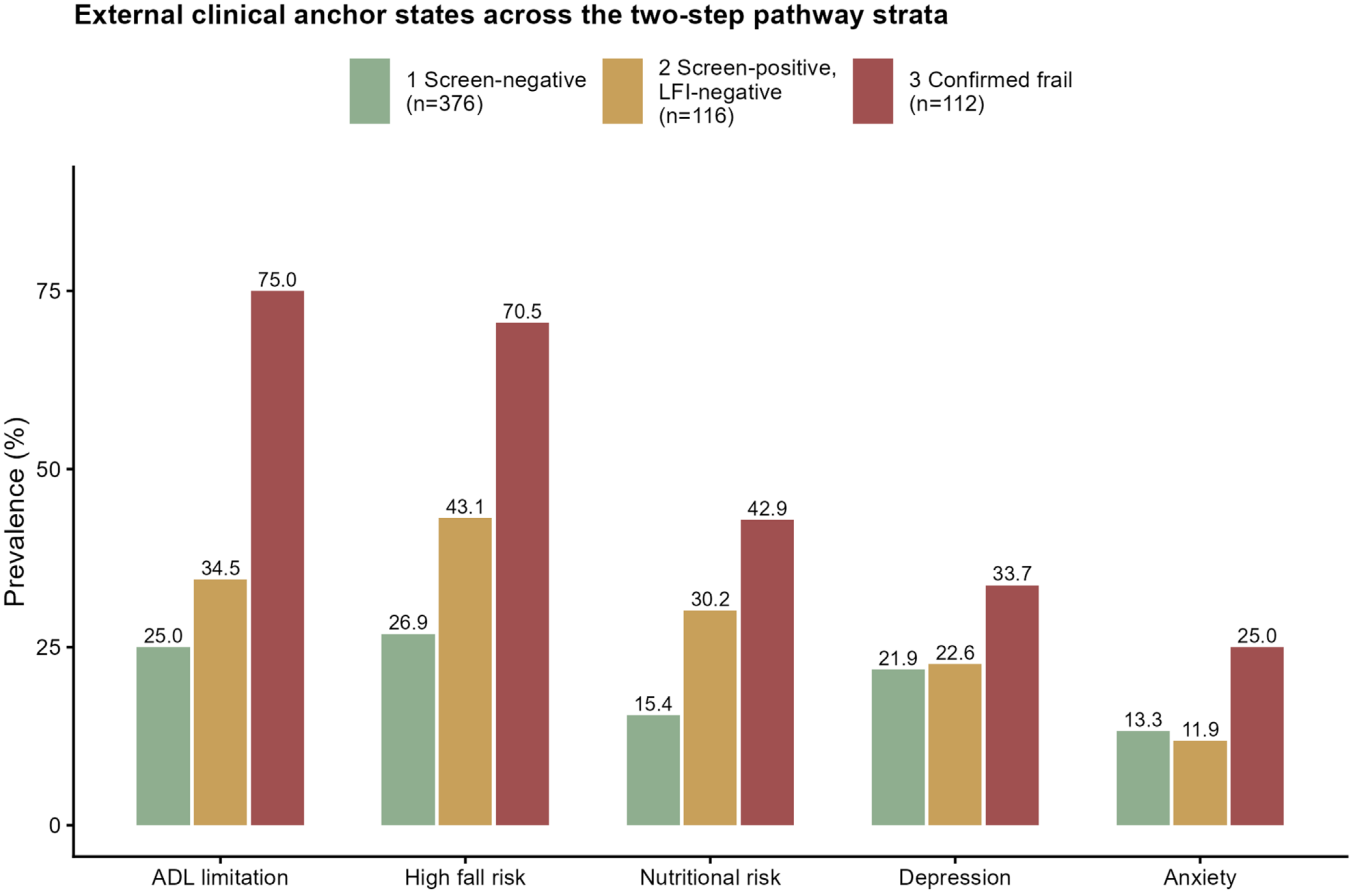
External clinical anchor states across the three two‐step pathway strata. ADL, activities of daily living.

In sex‐stratified analyses (Table S2, Figure S1), the OR rule missed zero frail patients in both sexes, although the pooled cutoffs yielded a positivity rate of 78.6% in women. With LFI ≥ 3.2 as the reference (Table S3), the OR rule achieved sensitivity of only 0.442. In the 600 patients with complete‐component data (Table S4), the OR rule again missed zero cases and spared 62.5% of the workload. Post hoc application of the pooled cutoffs across age and available clinical severity strata also yielded zero missed frailty cases, although workload saving was lower in older patients and those with more severe disease (Table S5).

## 4 Discussion

### 4.1 Principal Findings

In this cross‐sectional cohort of 604 patients with chronic liver disease, a two‐step sequential pathway—timed chair stands ≥ 16.55 s or grip strength ≤ 23.42 kg, with bedridden status counted as screen‐positive—identified all 112 patients with liver frailty index (LFI)‐defined frailty (sensitivity 1.000, 95% CI: 0.968–1.000; negative predictive value 1.000) while restricting the complete LFI to 37.7% of patients, thereby sparing 62.3% of full assessments (Table 3). This observed zero‐miss performance may partly reflect the shared components of the index tests and reference standard. Of the three LFI components, chair stands and grip strength carry the dominant weighting in the index (Lai et al. 2017; Wang et al. 2019); jointly they span the functional deficit spectrum required to reach an LFI of 4.5. Critically, the patients missed by each single component were entirely non‐overlapping—five frail patients were positive only on chair stands and nine only on grip strength—so the OR rule closes two complementary detection gaps. Standing balance, by contrast, showed poor pathway concordance (area under the receiver operating characteristic curve [AUC] 0.627) and is unsuitable as a stand‐alone initial screen; this honestly negative finding explains why it is reserved for the confirmatory second step.

The derived cutoffs are corroborated by external evidence. The chair‐stand threshold of 16.55 s approximates the > 15 s threshold embedded in the revised European sarcopenia consensus and foundational lower‐extremity performance batteries (Cruz‐Jentoft et al. 2019; Guralnik et al. 1994) and aligns with the > 15 s prognostic threshold reported in cirrhosis (Nguyen et al. 2023). The grip‐strength cutoff of 23.42 kg falls between the sex‐specific thresholds of the Asian Working Group for Sarcopenia (28 kg in men, 18 kg in women) (Chen et al. 2020) consistent with international normative data across the life course (Dodds et al. 2014, 2016). Finally, an exploratory grid search found that the maximal specificity attainable under a sensitivity = 1.0 constraint was 0.770—only marginally above the main‐strategy's 0.764 (Figure 4)—suggesting that the selected cutoffs sit near the feasible frontier rather than reflecting overfitting to this sample.

### 4.2 Comparison With Prior Work

Sequential two‐step screening has an established methodological lineage: in psychiatry, the ultra‐brief PHQ‐2 and GAD‐2 gate access to full diagnostic instruments (Kroenke et al. 2003, 2007; Levis et al. 2020); in geriatric medicine, simple performance measures have been systematically evaluated as first‐stage frailty screens (Clegg et al. 2015; Lee et al. 2017) and a pragmatic two‐step algorithm for sarcopenia and frailty was recently validated in community‐dwelling older adults (Aliberti et al. 2026). The present study applies this paradigm within the hepatology LFI framework, with its contribution centred on pathway efficiency.

Within hepatology, the closest prior studies addressed the assessment burden differently. Nguyen et al. established the independent prognostic value of the chair‐stand component but did not construct a screening pathway (Nguyen et al. 2023); the present work is the logical extension from prognostic association to pathway design. Thuluvath et al. used a brief patient‐reported PROMIS measure as a first‐stage screen in 81 transplant candidates (Thuluvath et al. 2024)—a questionnaire rather than an objective LFI‐component gate, in a small sample. The Tele‐Liver Frailty Index adapted the LFI to telemedicine (Wang et al. 2023) and the administrative frailty index derives frailty from coded health data (Calthorpe et al. 2026); both substitute the measurement setting rather than sequence the assessment. Geladari et al. compared five tools against the LFI in parallel and found only moderate inter‐tool agreement (*κ* 0.33–0.57), with the frail patients identified by different instruments not coinciding (Geladari et al. 2024); that discordance underscores the value of a pathway whose index tests are drawn from the same construct as the reference standard. Finally, the poor sensitivity of the questionnaire‐based SARC‐F in chronic liver disease (0.21 in a multicentre Japanese cohort) (Hanai et al. 2021) reinforces the necessity of objective performance tests as the first‐stage gate.

### 4.3 Clinical and Nursing Implications

The pathway was deliberately engineered for nursing‐led implementation. Both index tests are executable at the bedside within 2–3 min without specialised equipment, fitting established nurse‐led geriatric screening models (Lim et al. 2025) and aligning with precedent for bedside frailty assessment in hepatology (Tapper et al. 2015; Tandon et al. 2016). The 62.3% screen‐negative proportion suggests potential to reduce full LFI assessments and support guideline‐recommended universal screening (Lai, Tandon, et al. 2021), pending external validation. The stepwise external anchor gradient (Table 5, Figure 5)—rising activities of daily living limitation, fall risk, nutritional risk, depression, and anxiety from screen‐negative through screen‐positive/LFI‐negative to confirmed frail patients—supports a tiered management logic: a positive screen should trigger not only the complete LFI but also a comprehensive nursing assessment spanning function, falls, nutrition, and psychological status. Screening is clinically meaningful because frailty is modifiable: longitudinal change tracks mortality (Lai et al. 2020) frailty is associated with impaired quality of life (Lai et al. 2023) and exercise‐based and prehabilitation strategies show promise (Tandon et al. 2023; Li et al. 2024). One caveat is warranted: the pooled cutoffs were over‐sensitive in women (positivity 78.6%), whereas sex‐specific cutoffs (grip strength ≤ 18.58 kg, nearly identical to the Asian female threshold of 18 kg (Chen et al. 2020); chair stands ≥ 14.15 s; Table S2; Figure S1) maintained zero missed cases. Given documented sex differences in frailty and outcomes (Lai, Ganger, et al. 2021) sex‐specific first‐stage cutoffs merit prospective evaluation.

### 4.4 Methodological Defence

Because the index tests are components of the reference standard, all accuracy estimates are subject to incorporation bias (Ransohoff and Feinstein 1978); we therefore frame our findings as pathway and triage efficiency rather than independent diagnostic accuracy—a framing with direct precedent in the PHQ‐2/PHQ‐9 literature (Levis et al. 2020). This incorporation bias may partly account for the perfect sensitivity, NPV of 1.000, and absence of missed frail cases observed in the present cohort. Consequently, these performance metrics should not be interpreted as independent diagnostic accuracy estimates. The internal analyses assess stability but cannot remove incorporation bias. First, frozen‐cutoff split‐sample validation in five split‐sample analyses across four patient subsets (a temporal hold‐out and both odd–even directions) reproduced zero missed cases, supporting within‐cohort stability. Second, the external anchor gradient provides contextual support that pathway strata correspond to clinically meaningful functional burden, but does not constitute independent validation. Third, the boundary must be stated plainly: as quantified in Section 3.5, most screen‐negative patients were pre‐frail, and the highest LFI among them fell just below the frailty threshold. The pathway targets LFI‐defined frailty (LFI ≥ 4.5), not the full frailty spectrum. This is precisely the intended function of a first‐stage screen in geriatric two‐step models (Clegg et al. 2015) in which a negative first stage defers, rather than denies, further assessment; pre‐frail patients remain candidates for monitoring and preventive care. Exploratory subgroup analyses preserved zero missed frailty cases but showed lower efficiency in older patients and those with more severe disease (Table S5); the pooled cutoffs should therefore be regarded as provisional rather than universal pending external validation.

### 4.5 Limitations

Several limitations qualify these findings. First, the single‐centre, cross‐sectional design precludes any inference regarding prediction of future outcomes or longitudinal validation. Second, internal split‐sample validation is not true external validation; the cutoffs and the observed zero‐miss result require confirmation in prospective multicentre cohorts with different case mixes. Third, women numbered only 117, limiting the precision of sex‐specific estimates. Fourth, the pathway cannot rule out pre‐frailty, as detailed above. Fifth, the depression and anxiety instruments were not standardised by name in the source records, and component missingness, although rare (standing balance missing in 6 patients), was non‐zero. Sixth, device metrological information for the dynamometer—brand, model, nominal resolution, and calibration records—was not traceable from the archived records, which limits reproducibility assessment of the grip‐strength measurements. Seventh, assessors were not blinded to patients' clinical status. Finally, the LFI itself is an imperfect reference standard, albeit the most extensively validated instrument in this population (Lai et al. 2017; Jutras and Lai 2024) so pathway performance is conditioned on the LFI definition of frailty. All participants were hospitalised patients at a single centre, and the cut‐offs were derived and internally validated within the same cohort. These design features, together with the inherent incorporation bias arising from the use of LFI components as both index tests and reference standard, limit the interpretability of the accuracy estimates and preclude recommendation of the pathway for routine clinical practice until external validation in independent multicentre cohorts has been completed. Liver‐disease aetiology, fibrosis stage and reason for admission were not systematically retained, so subgroup effects across these factors could not be evaluated.

### 4.6 Future Directions

Prospective multicentre external validation is the immediate priority, including pre‐specified evaluation across age, sex, liver‐disease aetiology and disease stage. Longitudinal follow‐up should examine whether pathway‐defined strata are associated with hospitalisation and mortality. The pathway could also be extended into a broader multidimensional triage route incorporating nutrition and mood screening as further gates. Sex‐specific cutoffs warrant dedicated evaluation in adequately powered female cohorts, and implementation‐science studies should quantify time savings, nursing workload, and patient outcomes once the pathway is embedded into routine nursing workflows.

## 5 Conclusion

We developed and internally validated a nurse‐administered two‐step screening pathway built from the liver frailty index's (LFI) own rapid components. In 604 hospitalised patients with chronic liver disease, an either‐positive rule (timed chair stands ≥ 16.55 s or grip strength ≤ 23.42 kg; bedridden status counted as screen‐positive) identified all 112 patients with LFI‐defined frailty (sensitivity 1.000, 95% CI: 0.968–1.000; negative predictive value 1.000) while restricting the complete LFI to 37.7% of patients, sparing 62.3% of full assessments. This observed zero‐miss result reflected complementary capture (five frail patients positive only on chair stands, nine only on grip strength), was stable across 2000 bootstrap resamples and five split‐sample analyses across four patient subsets, and was accompanied by a stepwise external anchor gradient. The pathway's boundary is equally clear: it targets LFI‐defined frailty (LFI ≥ 4.5), not pre‐frailty, and its estimates reflect within‐pathway triage efficiency because the index tests are components of the reference standard.

In this cohort, the pathway could have spared nearly two‐thirds of hospitalised patients from complete LFI testing, with no observed missed LFI‐defined frailty cases. This finding does not establish independent diagnostic accuracy or clinical safety. Before routine adoption, the frozen cutoffs require prospective multicentre external validation in independent cohorts, adequately powered evaluation of sex‐specific thresholds, and implementation studies quantifying nursing workload and patient outcomes.

## Author Contributions

**Juan Wang:** conceptualization, data curation, formal analysis, investigation, project administration, software, validation, visualisation, writing – original draft. **Shan Ouyang:** data curation, formal analysis, investigation, methodology, writing – original draft. **Lihua Zheng:** data curation, investigation, validation. **Shuxian Jiang:** data curation, investigation, validation. **Na He:** investigation, methodology, validation, funding acquisition. **Lili Li:** conceptualization, methodology, resources, supervision, writing – review and editing.

## Funding

This study was supported by the Medical Scientific Research Foundation of Guangdong Province, China (Project No. A2023459).

## Disclosure

Reporting Method: This diagnostic accuracy study was reported in accordance with the STARD 2015 guideline.

Statistics: The authors have checked that this submission conforms, as applicable, to the Nursing Open statistical guidelines. Na He was responsible for the statistical review of the analysis. The authors affirm that the methods used in the data analyses are suitably applied to the data within the study design and context, and that the statistical findings have been implemented and interpreted correctly. The authors agree to take responsibility for ensuring that the choice of statistical approach is appropriate and is conducted and interpreted correctly.

Parent‐Cohort Publication Statement: The authors confirm that no other publication, abstract, preprint, thesis, or manuscript from this parent cohort has been published, posted, or submitted. The present manuscript is the first report from this cohort and contains no duplicate results.

## Ethics Statement

This study was approved by the Medical Ethics Committee of the Third Affiliated Hospital of Sun Yat‐sen University (approval number [2021]02‐060‐01; approval date 13 April 2021) and was conducted in accordance with the Declaration of Helsinki and applicable institutional and national requirements.

## Consent

Written informed consent was obtained from all participants before study procedures.

## Conflicts of Interest

The authors declare no conflicts of interest.

## Supporting information


**Table S1:** High‐sensitivity rule‐out thresholds for single step‐one tests (analysis set *n* = 604; frail = 112, reference standard LFI ≥ 4.5).
**Table S2:** Sex‐stratified screening performance of single components and the OR rule (reference standard LFI ≥ 4.5).
**Table S3:** Screening performance against the broader reference standard (LFI ≥ 3.2; frailty plus pre‐frailty).
**Table S4:** Complete‐component sensitivity analysis (patients with all three LFI components available, *n* = 600).
**Table S5:** Exploratory subgroup performance of the frozen OR‐rule pathway (analysis set *n* = 604; reference standard LFI ≥ 4.5).
**Figure S1:** Sex‐stratified receiver operating characteristic curves of grip strength for identifying frailty (LFI ≥ 4.5). Optimal cutoffs derived by the Youden index: men ≤ 23.42 kg; women ≤ 18.58 kg.

**Data S1:** STARD 2015 checklist.

## Data Availability

The participant‐level data supporting the findings of this study are not publicly available because of institutional ethics, privacy, and data‐governance restrictions. Requests for de‐identified data or analysis code may be directed to the responsible corresponding author and will be subject to institutional approval.
