# Supplementary material for: Two‐Step Sequential Screening for Frailty in Patients With Chronic Liver Disease: A Triage Efficiency Study of Liver Frailty Index Components
Source: Nurs Open. 2026 Oct 19;13(10):e70915. doi: 10.1002/nop2.70915 (PMC13679991; doi:10.1002/nop2.70915)
Supplement: Supplementary file 1 [file NOP2-13-e70915-s001.docx]

**STARD 2015 Checklist**

*Manuscript: Two-step sequential screening for frailty in patients with chronic liver disease: a triage efficiency study of Liver Frailty Index components*

*Reference: Bossuyt PM et al. STARD 2015: an updated list of essential items for reporting diagnostic accuracy studies. BMJ 2015;351:h5527.*

| **Section / Topic** | **No.** | **STARD 2015 item** | **Location / reporting status** |
| --- | --- | --- | --- |
| TITLE OR ABSTRACT | 1 | Identification as a study of diagnostic accuracy using at least one measure of accuracy (such as sensitivity, specificity, predictive values, or AUC) | Abstract Design and Results (p. 1, lines 9–18): diagnostic-accuracy design, sensitivity and NPV reported. |
| ABSTRACT | 2 | Structured summary of study design, methods, results, and conclusions | Structured Abstract (pp. 1–2, lines 7–31). |
| INTRODUCTION | 3 | Scientific and clinical background, including the intended use and clinical role of the index test | Introduction (pp. 2–3, lines 33–70): clinical background and intended triage role. |
|  | 4 | Study objectives and hypotheses | Introduction, final substantive paragraph (p. 3, lines 71–86). |
| METHODS — Study design | 5 | Whether data collection was planned before the index test and reference standard were performed (prospective study) or after (retrospective study) | Section 2.1 (p. 4, lines 91–97): cross-sectional design and consecutive enrolment. Timing of data-collection planning relative to testing is not explicitly stated. |
| Participants | 6 | Eligibility criteria | Section 2.1 (p. 4, lines 98–110): inclusion and exclusion criteria. |
|  | 7 | On what basis potentially eligible participants were identified (symptoms, results from previous tests, inclusion in registry) | Section 2.1 (p. 4, lines 91–97): patients with chronic liver disease undergoing clinical frailty assessment. |
|  | 8 | Where and when potentially eligible participants were identified (setting, location and dates) | Section 2.1 (p. 4, lines 91–97): single tertiary centre, hospitalised patients, August 2021–August 2025. |
|  | 9 | Whether participants formed a consecutive, random or convenience series | Section 2.1 (p. 4, lines 91–97): consecutive enrolment. |
| Test methods | 10a | Index test, in sufficient detail to allow replication | Section 2.2 (p. 5, lines 124–143): measurement procedures. Archived dynamometer details were unavailable; this is disclosed in Section 2.2 and Section 4.5. |
|  | 10b | Reference standard, in sufficient detail to allow replication | Section 2.3 (p. 6, lines 145–151): LFI calculation and frailty definition; component procedures in Section 2.2 (p. 5, lines 124–143). |
|  | 11 | Rationale for choosing the reference standard (if alternatives exist) | Introduction (p. 2, lines 46–56): rationale for LFI. Section 2.3 (p. 6, lines 145–166): reference standard and incorporation bias. |
|  | 12a | Definition of and rationale for test positivity cut-offs or result categories of the index test, distinguishing pre-specified from exploratory | Section 2.4 (p. 7, lines 173–186): Youden-derived cut-offs and OR rule; exploratory grid search in Section 2.6 (p. 8, lines 215–226). |
|  | 12b | Definition of and rationale for test positivity cut-offs or result categories of the reference standard, distinguishing pre-specified from exploratory | Section 2.3 (p. 6, lines 145–151): literature-based LFI ≥4.5; secondary reference LFI ≥3.2. |
|  | 13a | Whether clinical information and reference standard results were available to the performers or readers of the index test | Section 2.3, Blinding (p. 6, lines 167–171): no dedicated blinding; assessment order and assessor identity not documented. |
|  | 13b | Whether clinical information and index test results were available to the assessors of the reference standard | Section 2.3, Blinding (p. 6, lines 167–171); the LFI reference incorporates the index-test results (p. 6, lines 145–166). |
| Analysis | 14 | Methods for estimating or comparing measures of diagnostic accuracy | Section 2.6 (p. 8, lines 201–214): ROC/Youden, DeLong comparison, bootstrap and exact confidence intervals. |
|  | 15 | How indeterminate index test or reference standard results were handled | Section 2.1 (pp. 4–5, lines 98–122): inability to attempt chair stands because of bed rest triggers step-one positivity. LFI outcome was available for all 606 patients. |
|  | 16 | How missing data on the index test and reference standard were handled | Section 2.1 (pp. 4–5, lines 111–122): component missingness, available-case analysis, complete-component sensitivity analysis and no imputation. Section 3.1 (p. 9, lines 238–247). |
|  | 17 | Any analyses of variability in diagnostic accuracy, distinguishing pre-specified from exploratory | Section 2.6 (p. 8, lines 215–228): internal validation and prespecified versus post hoc analyses. Section 3.6 (p. 18, lines 354–361); Supplementary Tables S2–S5. |
|  | 18 | Intended sample size and how it was determined | Section 2.6 (p. 8, lines 226–228): no a priori sample-size calculation; all consecutive eligible patients assessed during the study period. |
| RESULTS — Participants | 19 | Flow of participants, using a diagram | Figure 1 (p. 10; caption, lines 249–250); Section 3.1 (p. 9, lines 238–247). |
|  | 20 | Baseline demographic and clinical characteristics of participants | Section 3.1 (pp. 10–11, lines 251–262); Table 1 (p. 11). |
|  | 21a | Distribution of severity of disease in those with the target condition | Table 1 (p. 11): Child–Pugh grade and decompensation by frailty status. Section 3.1 (pp. 10–11, lines 251–262). |
|  | 21b | Distribution of alternative diagnoses in those without the target condition | Table 1 (p. 11) characterises the non-frail group. Alternative liver-disease aetiologies were not retained; see Section 4.5 (p. 21, lines 461–462). |
|  | 22 | Time interval and any clinical interventions between index test and reference standard | Section 2.2 (p. 5, lines 124–125): single encounter. Section 2.3 (p. 6, lines 167–171): test order was not documented; intervening treatments are not separately reported. |
| Test results | 23 | Cross tabulation of the index test results (or their distribution) by the results of the reference standard | Section 3.3 (pp. 13–15, lines 294–324) and Table 3 (p. 14) report pathway counts and estimates. Frail-case overlap (98/5/9/0): p. 15, lines 312–320; Figure 3 (p. 14). A full 2×2 table is not displayed separately. |
|  | 24 | Estimates of diagnostic accuracy and their precision (such as 95% confidence intervals) | Section 3.2 (p. 12, lines 272–285), Section 3.3 (pp. 13–15, lines 294–324), and Section 3.4 (p. 16, lines 325–334); Tables 2–4 and Supplementary Table S5. Confidence intervals are reported for AUC and selected pathway estimates. |
|  | 25 | Any adverse events from performing the index test or the reference standard | Section 3.1 (p. 9, lines 246–247): no adverse events during the nurse-administered assessments. |
| DISCUSSION | 26 | Study limitations, including sources of potential bias, statistical uncertainty, and generalisability | Section 2.3 (p. 6, lines 152–171); Section 4.4 (pp. 20–21, lines 424–442); Section 4.5 (p. 21, lines 444–462). |
|  | 27 | Implications for practice, including the intended use and clinical role of the index test | Section 4.3 (pp. 19–20, lines 407–422); Section 5 (p. 22, lines 472–487). |
| OTHER INFORMATION | 28 | Registration number and name of registry | Not registered; no registry identifier. Registration status is stated in this checklist, not in the main manuscript. |
|  | 29 | Where the full study protocol can be accessed | No publicly accessible study protocol is provided. This is stated in this checklist, not in the main manuscript. |
|  | 30 | Sources of funding and other support; role of funders | Separate Title Page: Funding and Acknowledgments. The funder’s role is not specified in the supplied Title Page. |
