# Supplementary material for: Two‐Step Sequential Screening for Frailty in Patients With Chronic Liver Disease: A Triage Efficiency Study of Liver Frailty Index Components
Source: Nurs Open. 2026 Oct 19;13(10):e70915. doi: 10.1002/nop2.70915 (PMC13679991; doi:10.1002/nop2.70915)
Supplement: Supplementary file 2 [file NOP2-13-e70915-s002.docx]

**Supplementary Materials**

*Two-step sequential screening for frailty in patients with chronic liver disease: a triage efficiency study of Liver Frailty Index components*

Contents: Supplementary Tables S1–S5; Supplementary Figure S1

**Supplementary Table S1. High-sensitivity rule-out thresholds for single step-one tests (analysis set n = 604; frail = 112, reference standard LFI ≥ 4.5)**

| **Step-one strategy (target)** | **Cutoff** | **Sens** | **Spec** | **PPV** | **NPV** | **Full LFI required, n** | **Screen-positive, %** | **Missed frailty, n** | **Workload saved, %** |
| --- | --- | --- | --- | --- | --- | --- | --- | --- | --- |
| Timed chair stands (Sens ≥ 0.95) | ≥ 14.98 s | 0.964 | 0.886 | 0.659 | 0.991 | 164 | 27.2 | 4 | 72.8 |
| Timed chair stands (Sens ≥ 0.98) | ≥ 13.18 s | 0.982 | 0.783 | 0.507 | 0.995 | 217 | 35.9 | 2 | 64.1 |
| Grip strength (Sens ≥ 0.95) | ≤ 23.42 kg | 0.955 | 0.791 | 0.510 | 0.987 | 210 | 34.8 | 5 | 65.2 |
| Grip strength (Sens ≥ 0.98) | ≤ 28.83 kg | 0.982 | 0.551 | 0.332 | 0.993 | 331 | 54.8 | 2 | 45.2 |

*Note. Sens = sensitivity; Spec = specificity; PPV = positive predictive value; NPV = negative predictive value; LFI = Liver Frailty Index. Bedridden patients unable to perform timed chair stands were classified as screen-positive.*

**Supplementary Table S2. Sex-stratified screening performance of single components and the OR rule (reference standard LFI ≥ 4.5)**

| **Subgroup – test** | **n** | **Frail, n** | **AUC (95% CI)** | **Cutoff** | **Sens** | **Spec** | **PPV** | **NPV** | **Screen-positive, %** | **Missed, n** |
| --- | --- | --- | --- | --- | --- | --- | --- | --- | --- | --- |
| Men – Grip strength | 487 | 76 | 0.942 (0.913–0.965) | ≤ 23.42 kg | 0.934 | 0.881 | 0.592 | 0.986 | 24.6 | 5 |
| Men – Timed chair stands | 486 | 75 | 0.986 (0.973–0.997) | ≥ 16.85 s | 0.947 | 0.976 | 0.877 | 0.990 | 16.7 | 4 |
| Men – OR rule (16.55 s / 23.42 kg) | 487 | 76 | — | — | 1.000 | 0.854 | 0.559 | 1.000 | 27.9 | 0 |
| Women – Grip strength | 117 | 36 | 0.868 (0.796–0.926) | ≤ 18.58 kg | 0.944 | 0.704 | 0.586 | 0.966 | 49.6 | 2 |
| Women – Timed chair stands | 116 | 35 | 0.946 (0.904–0.976) | ≥ 14.15 s | 1.000 | 0.753 | 0.636 | 1.000 | 47.4 | 0 |
| Women – OR rule (16.55 s / 23.42 kg) | 117 | 36 | — | — | 1.000 | 0.309 | 0.391 | 1.000 | 78.6 | 0 |

*Note. AUCs and single-item cutoffs were derived within each sex stratum (Youden index); OR-rule rows apply the pooled cutoffs (16.55 s / 23.42 kg) to both sexes. The high screen-positive rate in women (78.6%) indicates the need for sex-specific cutoffs.*

**Supplementary Table S3. Screening performance against the broader reference standard (LFI ≥ 3.2; frailty plus pre-frailty)**

| **Test / strategy** | **n** | **LFI ≥ 3.2, n** | **AUC (95% CI)** | **Cutoff** | **Sens** | **Spec** | **PPV** | **NPV** | **Screen-positive, %** | **Missed, n** |
| --- | --- | --- | --- | --- | --- | --- | --- | --- | --- | --- |
| Grip strength | 604 | 504 | 0.867 (0.822–0.906) | ≤ 35.98 kg | 0.917 | 0.680 | 0.935 | 0.618 | 81.8 | 42 |
| Timed chair stands | 602 | 502 | 0.974 (0.961–0.985) | ≥ 8.30 s | 0.926 | 0.930 | 0.985 | 0.715 | 78.4 | 37 |
| OR rule (16.55 s / 23.42 kg) | 604 | 504 | — | — | 0.442 | 0.950 | 0.978 | 0.253 | 37.7 | 281 |
| OR rule, relaxed cutoffs (8.30 s / 35.98 kg) | 604 | 504 | — | — | 1.000 | 0.610 | 0.928 | 1.000 | 89.9 | 0 |

*Note. The recommended OR rule (16.55 s / 23.42 kg) excludes frailty (LFI ≥ 4.5) but not pre-frailty: sensitivity against LFI ≥ 3.2 was only 0.442. Relaxing the cutoffs achieved sensitivity 1.000 but required full LFI in 89.9% of patients, negating the efficiency gain. The two-step pathway is therefore positioned to rule out frailty, not the frailty spectrum.*

**Supplementary Table S4. Complete-component sensitivity analysis (patients with all three LFI components available, n = 600)**

| **Strategy** | **n** | **Frail, n** | **Sens** | **Spec** | **PPV** | **NPV** | **Full LFI required, n** | **Screen-positive, %** | **Missed, n** | **Workload saved, %** |
| --- | --- | --- | --- | --- | --- | --- | --- | --- | --- | --- |
| OR rule (16.55 s / 23.42 kg) | 600 | 110 | 1.000 | 0.765 | 0.489 | 1.000 | 225 | 37.5 | 0 | 62.5 |

*Note. Results were consistent with the primary analysis set (n = 604), indicating that available-case handling of missing components did not materially affect the findings.*

**Supplementary Table S5. Exploratory subgroup performance of the frozen OR-rule pathway (analysis set n = 604; reference standard LFI ≥ 4.5)**

| **Subgroup** | **n** | **Frail, n (%)** | **Sensitivity (95% CI)** | **Specificity** | **NPV (95% CI)** | **Full LFI required, n (%)** | **Workload saved, %** | **Missed, n** |
| --- | --- | --- | --- | --- | --- | --- | --- | --- |
| Age <40 years | 141 | 15 (10.6) | 1.000 (0.782–1.000) | 0.817 | 1.000 (0.965–1.000) | 38 (27.0) | 73.0 | 0 |
| Age 40–59 years | 324 | 42 (13.0) | 1.000 (0.916–1.000) | 0.787 | 1.000 (0.984–1.000) | 102 (31.5) | 68.5 | 0 |
| Age ≥60 years | 139 | 55 (39.6) | 1.000 (0.935–1.000) | 0.607 | 1.000 (0.930–1.000) | 88 (63.3) | 36.7 | 0 |
| Child–Pugh A | 123 | 17 (13.8) | 1.000 (0.805–1.000) | 0.792 | 1.000 (0.957–1.000) | 39 (31.7) | 68.3 | 0 |
| Child–Pugh B | 242 | 42 (17.4) | 1.000 (0.916–1.000) | 0.790 | 1.000 (0.977–1.000) | 84 (34.7) | 65.3 | 0 |
| Child–Pugh C | 239 | 53 (22.2) | 1.000 (0.933–1.000) | 0.720 | 1.000 (0.973–1.000) | 105 (43.9) | 56.1 | 0 |
| No ascites | 280 | 40 (14.3) | 1.000 (0.912–1.000) | 0.783 | 1.000 (0.981–1.000) | 92 (32.9) | 67.1 | 0 |
| Ascites | 324 | 72 (22.2) | 1.000 (0.950–1.000) | 0.746 | 1.000 (0.981–1.000) | 136 (42.0) | 58.0 | 0 |
| No recorded decompensation | 246 | 33 (13.4) | 1.000 (0.894–1.000) | 0.793 | 1.000 (0.978–1.000) | 77 (31.3) | 68.7 | 0 |
| Recorded decompensation | 358 | 79 (22.1) | 1.000 (0.954–1.000) | 0.742 | 1.000 (0.982–1.000) | 151 (42.2) | 57.8 | 0 |

*Note. The pooled cutoffs (timed chair stands ≥16.55 s or grip strength ≤23.42 kg) were applied unchanged; no subgroup-specific cutoffs were re-estimated. Recorded decompensation was defined as the presence of ascites, hepatic encephalopathy, or gastrointestinal bleeding in the source record. Sensitivity and NPV are shown with Clopper–Pearson exact 95% confidence intervals. NPV was 1.000 in all strata. This analysis was post hoc and exploratory. CI = confidence interval; LFI = Liver Frailty Index; NPV = negative predictive value.*

**Supplementary Figure S1**

*Supplementary Figure S1. Sex-stratified receiver operating characteristic curves of grip strength for identifying frailty (LFI ≥ 4.5). Optimal cutoffs derived by the Youden index: men ≤ 23.42 kg; women ≤ 18.58 kg.*
